# Association between weight change and risk of liver fibrosis in adults with type 2 diabetes

**DOI:** 10.7189/jogh.13.04138

**Published:** 2023-10-20

**Authors:** Pan Ke, Minzhi Xu, Jie Feng, Qingfeng Tian, Yan He, Kai Lu, Zuxun Lu

**Affiliations:** 1 Department of Social Medicine and Health Management, School of Public Health, Tongji Medical College, Huazhong University of Science and Technology, Wuhan, Hubei, China; 2 School of Public Health, Zhengzhou University, Zhengzhou, Henan, China; 3 Tongji Hospital Affiliated to Tongji Medical College, Huazhong University of Science and Technology, Wuhan, Hubei, China

## Abstract

**Background:**

Liver fibrosis plays a key role in the progression of non-alcoholic fatty liver disease to cirrhosis. Considering weight change is known to be closely associated with increased risk of liver fibrosis, we aimed to address a gap in evidence regarding the existence of this association in patients with type 2 diabetes (T2D).

**Methods:**

We included data on 622 T2D patients and 1618 non-T2D participants from the 2017-2018 cycle of the National Health and Nutrition Examination Survey (NHANES). We assessed liver fibrosis by the median values of liver stiffness measurement (LSM). According to the participants' body mass index (BMI) at age 25 (early adulthood), 10 years prior (middle adulthood), and at the 2017-2018 cycle (late adulthood), we categorised weight change patterns into stable non-obese, weight loss, weight gain, and stable obese. We applied logistic regression to association analysis and used population attributable fraction (PAF) to analyses hypothetical prevention regimens.

**Results:**

The prevalence of liver fibrosis was higher in T2D patients (23.04%) than in non-T2D participants (6.70%), while weight change was associated with a greater risk of fibrosis in the former compared to the latter group. Compared with T2D patients in the stable non-obese group, stable obese individuals from 10 years prior to the 2017-2018 cycle had the highest risk of developing liver fibrosis, corresponding to an adjusted odds ratio (aOR) of 3.13 (95% confidence interval = 1.84-5.48). Absolute weight change patterns showed that the risk of liver fibrosis was highest (aOR = 2.94) when T2D patients gained at least 20 kg of weight from 10 years prior to 2017-2018 cycle.

**Conclusions:**

Obesity in middle and late adulthood is associated with an increased risk of T2D complicated with liver fibrosis.

Non-alcoholic fatty liver disease (NAFLD) has become the most common form chronic liver disease in the USA [1]; it is closely relate to obesity, ectopic fat deposition, insulin resistance, high fructose, cholesterol intake, and genetic mutation [2,3]. About 38% of NAFLD patients will develop liver fibrosis or even liver cirrhosis and liver cancer [4], making early identification of liver fibrosis and its risk factors crucial.

The degree of fibrosis is the best predictor of future NAFDL-related clinical outcomes [5]. Notably, early identification of patients with liver fibrosis and risk factors can potentially prevent progression to decompensated cirrhosis, hepatocellular carcinoma (HCC), as well as liver-related and non-liver-related death [6]. Although liver biopsy is the gold standard for assessing liver fibrosis staging [7], it is expensive and invasive, with poor patient compliance. Current guidelines recommend the use of non-invasive tests to evaluate liver lesions, such as ultrasound transient elastography (TE) to screen for liver stiffness [8].

Weight change reflects health trajectories over an individual's life course. Previous studies have shown that weight gain from adolescence to mid or late adulthood is associated with increased morbidity and mortality risk from diabetes, cardiovascular disease (CVD), cancer, and all-cause mortality [9,10]. Surprisingly, only four studies examined the association of weight change in adulthood with NAFLD [11-14].

Previous studies have shown that type 2 diabetes (T2D) is closely related to the occurrence and progression of liver fibrosis [15,16], based on a pathological mechanism through which high blood glucose potentially has a direct toxic effect on liver cells, leading to liver cell damage and death. Although the association between weight change and liver fibrosis risk has been explored, it is unclear whether this risk also exists in patients with T2D, who tend to have a longer disease course and go through dynamic changes in weight. We aimed to address this gap by investigating the prevalence of liver fibrosis in T2D and non-T2D participants and comparing the relationship between weight change and the risk of liver fibrosis in these two groups.

## METHODS

### Study population

We conducted a cohort study using data from the 2017-2018 cycle of National Nutritional Health Survey (NHANES), which specifically assessed liver fibrosis with TE. We defined T2D according to any of the following criteria from the American Diabetes Association (ADA) [17]:

1. Self-reported diagnosis of diabetes;
2. Haemoglobin A1c (HbA1c) level ≥6.5% (48 mmol/mol);
3. Fasting plasma glucose (FPG) ≥126 mg/dL.

**Figure 1:**
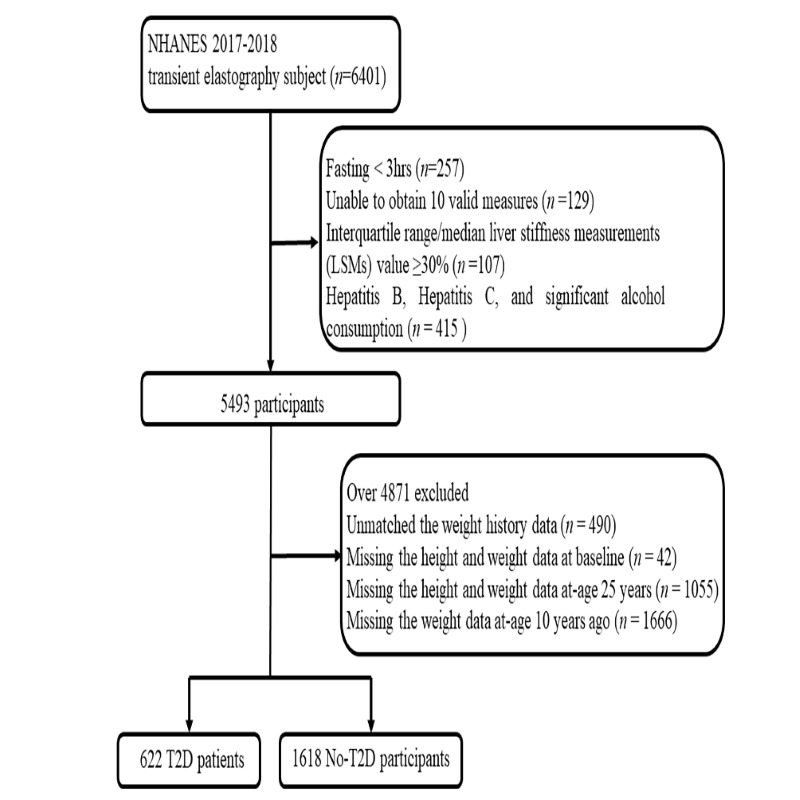
Data exclusion process.

We defined T2D duration as the cumulative period from the age at which an individual was first diagnosed with T2D to the 2017-2018 cycle survey. Overall, 6401 participants underwent TE during the 2017-2018 cycle; we excluded 493 who did not fulfil the fasting requirement (n = 257), had <10 valid measurements (n = 129), or had an interquartile range/median liver stiffness measurements (LSM) value ≥30 (n = 107). We also excluded individuals positive for hepatitis B virus surface antigen and hepatitis C virus surface antibody RNA, and participants with significant alcohol consumption (n = 453). We estimated alcohol consumption based on self-reported data on the amount and frequency of alcohol use within the previous year. It was considered significant an individual consumed >1 drink per day (for females) and >2 drinks per day (for males = on average). We then excluded missing values for body mass index (BMI) (2017-2018 cycle, 10 years prior, age 25). After merging data, we could no longer match data on participants with information on drug use and dietary intake, because they were indirectly excluded when merging data on weight and diabetes. Finally, we enrolled a total of 2240 participants, 622 of whom were T2D patients and 1618 of whom were no-T2D (**Figure 1**). We followed the STROBE statement in conducting the study [43].

### Exposure

Respondents were asked to self-report their weight and height at age 25 (early adulthood) and their weight 10 years prior (mid-adulthood) the 2017-2018 cycle, while these values were measured via physical examination for the exact 2017-2018 cycle (late adulthood). We converted the said values to kgs (for weight) and m (for height) to calculate BMI (kg/m^2^) for each corresponding period.

We created patterns of weight change for three times intervals for BMI: age 25 to 10 years prior, age 25 to 2017-2018 cycle, and 10 years prior to 2017-2018 cycle [9]. We used four categories of BMI change patterns: stable non-obese (age 25 < 30 kg/m^2^ and 10 years prior <30 kg/m^2^), losing (age 25 ≥ 30 kg/m^2^ and 10 years prior <30 kg/m^2^), gaining (age 25 < 30 kg/m^2^ and 10 years prior ≥30 kg/m^2^), and stable obese (age 25 ≥ 30 kg/m^2^ and 10 years prior ≥30 kg/m^2^). We calculated the weight change categories for age 25 to 2017-2018 cycle and 10 years prior to 2017-2018 cycle as above [10]. We also divided the absolute weight change within each time interval into five groups: weight loss of at least 2.5 kg, weight change within 2.5 kg (reference group), weight gain of at least 2.5 kg but less than 10.0 kg, and weight gain of at least 10.0 kg but less than 20.0 kg, andf weight gain of at least 20.0 kg [9,18].

### Outcome

FibroScan model 502 V2 Touch (Echosens^®TM^, Echosens, Paris, France) was performed, equipped with a medium (M) or extra-large (XL) probe. The M probe was used initially unless the machine indicated use of the XL probe. LSM scores were measured to detect liver fibrosis. A median LSM≥8.2 kPa was considered the threshold for significant fibrosis (≥F1), while values ≥9.7 kPa and ≥13.6 kPa were considered the threshold for advanced fibrosis (F2) and cirrhosis (F3), respectively [8].

### Covariates

Covariate information was obtained at 2017-2018 cycle by physical examination, laboratory tests, and questionnaires. Sociodemographic characteristics including sex (males, females), age, education (high school below, high school education or equivalent, college degree or above), and marital status (married, divorce). Smoking, drinking, and physical activity (PA) were obtained through self-reporting. Physical examination measured systolic blood pressure (SBP), diastolic blood pressure (DBP), waist circumference (WC). Laboratory-checked levels of HbA1c, total cholesterol (TC), total triglycerides (TG), platelet count, alanine aminotransferase (ALT), aspartate aminotransferase (AST), gamma-glutamyl transpeptidase (GGT), creatinine, albumin, globulin, high-sensitivity C-reactive protein (hs-CRP), and other assays have been detailed elsewhere [19]. Individuals’ comorbidities – chronic diseases, such as dyslipidaemia (DLP), hypertension (HTN), CVD (congestive heart failure, coronary heart disease, heart attack, stroke), and cancer, were obtained by physical examination, laboratory tests, or self-report [20].

### Statistical analysis

We applied appropriate sampling weight analysis in our analysis. We expressed continuous variables as means with standard deviations (SDs) or medians with interquartile ranges (IQRs), testing for differences between groups using an independent sample *t*-test or non-parametric test. We presented categorical variables as numbers and percentages, and tested for differences between groups using χ^2^ test. We applied bivariate logistic regression to evaluate the association of weight change patterns and absolute weight change on the presence of fibrosis, which we reported through odds ratios (ORs) and 95% confidence intervals (CIs). We performed subgroup analyses based on sex, age (2017-2018 cycle), and T2D duration. Additionally, we employed a restricted cubic spline regression model with three knots (5th, 50th, and 95th percentiles) to estimate the linear dose-response relationship between BMI at three time points, and with absolute weight change and liver fibrosis risk.

We calculated population attributable fraction (PAF) [10,12] to estimate the percentage of liver fibrosis cases that could be prevented under four hypothetical scenarios (Table S7 in the **Online Supplementary Document**).The category-specific attributable fraction is the percentage of liver fibrosis cases that would be eliminated if individuals in that weight change category were moved to another lower-risk category, assuming causation. We also performed sensitivity analyses to evaluate the stability of the results.

We performed all statistical analyses in R, version 4.10 (R Core Team, Auckland, New Zealand) and Stata, version 14.0 (StataCorp, College Station, TX, USA), with the Stata package “punafcc” for calculating PAF. We considered a two-tailed value of *P* < 0.05 as statistically significant, but set the test level for interaction effects at *P* < 0.10.

## RESULTS

### Participants characteristics

LSM, CAP, age, BMI, WC, DBP, ALT, GGT, LDH, albumin to globulin ratio, hs-CRP, HbA1c, TC, TG values, and the prevalence of HTN, DLP, CVD, cancer were higher in T2D compared to non-T2D participants; this difference was statistically significant (Table S1 in the **Online Supplementary Document**).

### Prevalence of liver fibrosis

Among 622 T2D patients, there were 131 with any degree of liver fibrosis, with an overall weighted prevalence (wp) of 23.04% (95% CI = 17.86-29.20). In this group, there were 39 patients with grade 1 (wp = 7.21%; 95% CI = 4.49-11.38), 51 with grade 2 (wp = 8.44%; 95% CI = 5.58-12.57), and 41 with grade 39 liver fibrosis (wp = 7.39%; 95%CI = 4.24-12.59). Among 1618 non-T2D participants, there were 150 patients with any degree of liver fibrosis (wp = 6.70%; 95% CI = 5.10-8.16), among whom 57 had grade 1 (wp = 2.05%; 95% CI = 1.35-3.10), 55 had grade 2 (wp = 2.55%; 95% CI = 1.69-3.83), and 38 had grade 3 liver fibrosis (wp = 2.10%; 95% CI = 1.15-3.81). The prevalence of liver fibrosis was significantly higher in T2D patients than in non-T2D participants (Table S2 in the **Online Supplementary Document**).

### Relationship of weight change patterns with liver fibrosis

**Figure 2:**
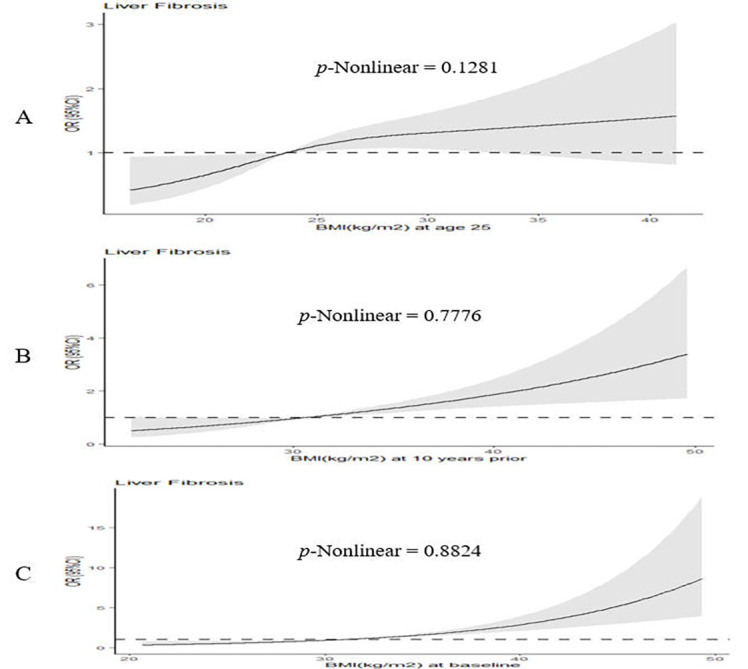
Dose-response curves of BMI at three time points associated with liver fibrosis risk among T2D patients.

After adjusting for all covariates, the risk of liver fibrosis was highest for the stable obese group in both T2D patients and non-T2D participants (**Table 1** and **Table 2**). However, the risk of liver fibrosis was higher in stable obese individuals with T2D (OR = 3.86; 95% CI = 1.78-16.53) than in their non-T2D peers (OR = 3.46; 95% CI = 1.46-8.18). T2D patients and the stable obese group of non-T2D participants had the highest risk in the 10 years period to 2017-2018 cycle; however, this risk was higher in T2D patients (OR = 3.13; 95%CI = 1.84-5.48) compared to the non-T2D participants (OR = 2.63; 95%CI = 1.40-4.42) (Tables S3-S4 in the **Online Supplementary Document**). However, weight loss was rare, and the association was not statistically significant among T2D patients. When evaluating the weight status at each time point (**Figure 2**), we found that the association of BMI with fibrosis risk at age 25 years, 10 years prior, and at 2017-2018 cycle was linear among T2D patients.

**Table 1:** Association of weight changes with liver fibrosis risk among T2D patients

	Weight change patterns			
**Liver fibrosis**	**Stable non-obese (reference)**	**Weight loss, OR (95%CI)**	**Weight gain, OR (95%CI)**	**Stable obese, OR (95%CI)**
Events/total	24/203	8/70	83/292	16/51
Model 1*	1.00	0.88 (0.38-2.05)	2.96 (1.80-4.87)	3.46 (1.65-7.07)
Model 2†	1.00	0.85 (0.36-1.99)	3.13 (1.84-5.00)	3.41 (1.62-7.18)
Model 3‡	1.00	0.45 (0.34-6.16)	3.24 (1.24-8.05)	3.86 (1.78-16.53)

BMI – body mass index, OR – odds ratios, CI –confidence interval, T2D – type 2 diabetes

*Crude model.

†Adjusted for sex and age.

‡Adjusted for sex, age, education, marital status, smoking, alcohol consumption, physical activity, SBP, DBP, WC, ALT, AST, GGT, LDH, hs-CRP, Uric acid to creatinine ratio, albumin to globulin ratio TC, TG, platelet count, dyslipidaemia, hypertension, CVD, and cancer.

**Table 2:** Association of weight changes with liver fibrosis risk among no-T2D participants

	Weight change patterns			
**Liver fibrosis**	**Stable non-obese (reference)**	**Weight loss, OR (95%CI)**	**Weight gain, OR (95%CI)**	**Stable obese, OR (95%CI)**
Events/total	57/940	8/117	76/512	9/49
Model 1*	1.00	1.14 (0.53-2.46)	2.70 (1.88-3.88)	3.49 (1.61- 7.54)
Model 2†	1.00	1.04 (0.48-2.25)	2.86 (1.98-4.13)	3.68 (1.68-8.06)
Model 3‡	1.00	0.80 (0.33-1.93)	3.23 (2.12-4.93)	3.46 (1.46-8.18)

BMI – body mass index, OR – odds ratios, CI –confidence interval, T2D – type 2 diabetes

*Crude model.

†Model 2: Adjusted for sex and age.

‡Model 3: Adjusted for sex, age, education, marital status, smoking, alcohol consumption, physical activity, SBP, DBP, WC, ALT, AST, GGT, LDH, hs-CRP, Uric acid to creatinine ratio, albumin to globulin ratio TC, TG, platelet count, dyslipidaemia, hypertension, CVD, and cancer.

### Association of absolute weight change with liver fibrosis among T2D patients

Since the relationship between weight change and the risk of liver fibrosis was greater in T2D patients, we performed subgroup analyses of absolute weight change and demographic characteristics in T2D patients. When classified into categories (Table S5 in the **Online Supplementary Document**), the OR for liver fibrosis in the extreme weight gain (weight gain ≥20 kg) group were 2.94 (95% CI = 1.24-7.07) from 10 years prior to 2017-2018 cycle among T2D patients. However, weight loss of at least 2.5kg was not associated with increased liver fibrosis risk. The relationship between absolute weight change from 10 years prior to 2017-2018 cycle, with a nonlinear risk for liver fibrosis risk (*P* = 0.031) (Figure S1, panel C in the **Online Supplementary Document**).

### Subgroup analysis among T2D patients

We found an association between weight change patterns and liver fibrosis within males (OR = 3.64; 95% CI = 1.79-7.42) and patients younger than 65 years (OR = 3.56; 95% CI = 1.59-7.95), while T2D patients maintained obesity from 10 years ago to 2017-2018 cycle (Table S6 in the **Online Supplementary Document**). Individuals who have been obese from age 25 to the 2017-2018 cycle and had a T2D duration of more than 10 years had a OR of 4.88 (95% CI = 1.38-17.35). We found an interactive effect of T2D duration and weight change when BMI changed from age 25 to 2017-2018 cycle (*P* = 0.037).

### PAF analysis among T2D patients

In the weight loss scenario, 60.71% (95% CI = 39.75-64.83) of liver fibrosis events could have been if those who gained weight from early adulthood had lost weight in late adulthood (Table S7 in the **Online Supplementary Document**). With weight maintenance, 58.55% (95% CI = 37.05-72.71) of liver fibrosis events could have been if those who gained weight remained non-obese in adulthood. In partial prevention regimens, 53.70% (95% CI = 30.24-69.28) of liver fibrosis events could have been prevented if the T2D patients remained stable and non-obese (BMI<30 kg/m^2^) throughout life. However, as there were only 39 liver fibrosis cases with BMI<25 kg/m^2^ across the three lifespans in our analysis, we did not observe a protective effect of the comprehensive preventive regimen.

### Sensitivity analysis among T2D patients

Risk of significant liver fibrosis and cirrhosis was highest in stable obesity from 10 years prior to 2017-2018 cycle, while the risk of advanced fibrosis was highest in stable obesity from age 25 to 2017-2018 cycle survey (Table S8 in the **Online Supplementary Document**).

## DISCUSSION

### Main findings

We found tha the prevalence of liver fibrosis was higher in T2D patients than in non-T2D participants and that weight change was more associated with fibrosis risk in T2D patients than in non-T2D participants. We also observed the highest risk of liver fibrosis in stable obese individuals from mid to late adulthood among T2D patients. These findings suggest that enhanced weight management in midlife could contribute to reduce the risk of liver fibrosis in adults with T2D.

### Interpretation of the results

Previous studies have suggested that the mechanisms underlying the deleterious effects of weight change over certain lifespans may differ [9,21]. For example, in early adulthood, weight gain primarily results from the accumulation of fat mass [22-24], while in late adulthood, it is usually attributed to a decrease in lean body mass and an increase in fat mass [25,26]. Our results are therefore in line with previous studies showing that obesity in midlife exacerbates lipid deposition [27]. Imbalances in lipid metabolism lead to the formation of lipotoxic intermediates that contribute to cellular stress (ie, oxidative stress and endoplasmic reticulum stress), inflammasome activation, and apoptotic cell death, followed by inflammation, tissue regeneration, and fibrogenesis [28]. Previous evidence suggests that mild fibrosis (F1), considered an early stage of NAFLD, mostly does not progress to cirrhosis [29]. However, in the setting of obesity and T2D, many patients with fibrosis may be at risk for being “rapid progressors” to more severe liver disease [30,31]. We found that 23.04% of T2D patients had liver fibrosis, which supports the evidence that obese and diabetic patients are at highest risk and need more aggressive screening.

Besides obesity status, more research is now focusing on the association between weight change and health outcomes, as weight change is common throughout adulthood [32,33]. A large prospective cohort study found that obesity and weight gain were independently associated with an increased risk of liver fibrosis progression [34]. We also found that the effect of weight gain was generally stronger from young to late adulthood than from young to mid-adulthood and mid-adult to late adulthood. Weight gain in early adulthood was more strongly associated with adverse metabolic markers (e.g. adiponectin, C-peptide, HbA1c, and GGT) than weight gain in late adulthood [35]. Excessive free fatty acid transport to the liver and skeletal muscle leads to increased intrahepatic triglycerides and leads to hepatic fat accumulation, which further contributes to liver fibrotic lesions [36]. We found that extreme weight gain (≥20 kg) in the 10 years prior to 2017-2018 cycle was associated with a 194% increased liver fibrosis risk, but found no such association for low to moderate weight gain (ranging from 2.5 kg to 20 kg) or weight loss of at least 2.5 kg. We also found that only absolute weight change from 10 years prior to 2017-2018 cycle exhibited a J-shaped nonlinear association with liver fibrosis. Although the underlying mechanisms require further investigation, our findings suggest that controlling weight gain at middle age would be an effective strategy to reduce the risk of future liver fibrosis.

In contrast to our study, Ciardullo et al. found a significant effect of body fat distribution on liver fibrosis in women rather than in men [37]. A recent meta-analysis showed that men are at a higher risk of developing NAFLD compared to women [38]. Although we are unable to determine the reason for the differences between studies, sex hormones might play a role in the progression of liver fibrosis [39]. Previous studies have shown that with age, the number and volume of individual hepatocytes decreases as hepatic blood flow declines, which may increase liver stiffness [40]. Our subgroup analysis showed that individuals with T2D duration of more than 10 years who remained obese throughout adulthood had a higher risk of developing liver fibrosis than those 25 to 10 years prior and 10 years prior to 2017-2018 cycle. Considering the wide 95% CI (1.38-17.35), this might be related to the small number of events. This finding suggests that the longer the duration of T2D, the more obesity begins at a young age, and the higher the risk of liver fibrosis.

We also employed a PAF analysis to explore the potential impact of weight loss interventions and preventive measures against weight gain.

Sensitivity analysis showed that stable obesity in adulthood resulting in the highest risk of advanced liver fibrosis, which may be related to the smaller sample size. However, wo found that weight control is beneficial in reducing liver fibrosis risk in T2D patients.

### Strengths and limitations

Our study had several strengths. It is the first to explore the association of weight change with liver fibrosis risk in adults with T2D using data from the NHANES. Our PAF analysis suggest that starting weight control in midlife is beneficial for reducing and preventing liver fibrosis risk.

Our study also had some limitations. We used recalled and self-reported weight data from 25 age years and 10 years prior, making misclassification bias possible. However, several studies suggest that recalling early life weight may be a valid indicator for use in life course epidemiological analyses [41,42]. We did not apply other non-invasive markers such as Fibrosis 4 score (FIB-4) to diagnose liver fibrosis because there were only 14 liver fibrosis events, which may lead to many liver fibrosis events being missed if this index was used. We also did not include drug treatments such as Glucagon-like peptide-1 receptor agonist (GLP-1) in the analysis due to unmatched data. Moreover, we did not adjust for covariates such as income or dietary intake in the regression models, as these variables could not be matched after through the inclusion and exclusion criteria. Finally, we did not evaluate changes in other obesity-related markers such as WC and fat mass in relation to liver fibrosis due to the lack of data at different time points.

## CONCLUSIONS

We found that stable obesity in midlife and weight gain from young age were associated with an increased liver fibrosis risk in adults with T2D. Future studies are needed to determine the relationship between changes in body composition in adulthood with liver fibrosis.

## Additional material


Online Supplementary Document
