# Supplementary material for: Association between weight change and risk of liver fibrosis in adults with type 2 diabetes
Source: J Glob Health. 2023 Oct 20;13:04138. doi: 10.7189/jogh.13.04138 (PMC10586795; doi:10.7189/jogh.13.04138)
Supplement: Online Supplementary Document [file jogh-13-04138-s001.pdf]

Supplementary Tables and Figures Legend

Table S1. Features of the study population

Table S2. Weighted prevalence of liver fibrosis among participants

Table S3. Association of weight changes across three lifespans with liver fibrosis risk among T2D patients

Table S4. Association of weight changes across three lifespans with liver fibrosis risk among no-T2D participants

Table S5. Association of absolute weight change patterns with liver fibrosis risk among T2D patients

Table S6. Subgroup analysis of weight change patterns and liver fibrosis risk among T2D patients

Table S7. Population attributable fractions (PAF) for population counterfactuals of fibrosis among T2D patients

Table S8. Sensitivity analysis of the association between weight change and liver fibrosis in T2D patients

Figure S1. Dose-response curves of absolute weight change associated with liver fibrosis risk among T2D patients

Table S1. Features of the study population

Participant characteristics	T2D(*n*=622)	No-T2D(*n*=1618)	*p*-value
LSM (kPa, mean ± SD)	7.07±4.45	5.79±4.89	<0.001
CAP (dB/m, mean ± SD)	300.75±58.68	264.32±57.39	<0.001
Age(years, mean ± SD)	66.06±8.75	64.09±8.99	<0.001
Males, (*n*, %)	343(55.14)	793(49.01)	0.009
Married, (*n*, %)	373(59.97)	889(54.94)	0.032
College degree or above (*n*, %)	341(54.82)	910(56.24)	0.631
Disease duration (years) [M, (P_{25}, P_{75})]	11.50(5, 20)	-	-
Non-Hispanic White, (*n*, %)	204(32.80)	662(40.92)	<0.001
BMI $_{2017\text{ - }2018\text{ cycle}}$ (kg/m^2, mean ± SD)	31.86±6.93	28.58±6.18	<0.001
BMI $_{\text{at 10 years prior the 2017 - 2018 cycle}}$ (kg/m^2, mean ± SD)	31.75±6.92	27.74±5.72	<0.001
BMI $_{\text{at age 25 years}}$ (kg/m^2, mean ± SD)	24.65±5.81	22.95±4.14	<0.001
WC (cm, mean ± SD)	108.94±15.35	99.87±14.64	<0.001
SBP (mmHg, mean ± SD)	135.76±20.87	133.87±20.38	0.051
DBP (mmHg, mean ± SD)	70.05±14.40	73.96±13.19	<0.001
Current smoking, (*n*, %)	294(47.27)	767(47.40)	0.954

Alcohol consumption (cup/d, mean ± SD)	1.69±0.23	1.71±0.27	0.094
Moderate PA, (*n*, %)	227(36.50)	395(24.41)	0.052
ALT (U/L, mean ± SD)	22.67±16.27	21.20±13.65	0.015
AST (U/L, mean ± SD)	22.00±13.69	22.33±10.75	0.279
GGT (IU/L, mean ± SD)	37.73±45.93	32.28±42.06	0.006
LDH (IU/L, mean ± SD)	166.45±36.17	161.33±41.68	0.005
Albumin to globulin ratio (mean ± SD)	1.35±0.24	1.36±0.24	<0.001
Uric acid to creatinine ratio (mean ± SD)	6.39±2.10	6.25±1.72	0.059
hs-CRP (mg/L, mean ± SD)	5.59±10.15	3.72±7.08	<0.001
HbA1c (%)	7.32±1.49	5.63±0.35	<0.001
TC (mg/dl, mean ± SD)	198.72±41.68	179.05±46.94	<0.001
TG (mg/dl, mean ± SD)	173.15±129.45	139.17±108.65	<0.001
Platelet count (10^9/L)	233.01±64.26	230.62±60.85	0.413
Combined HTN (*n*, %)	215(34.57)	595(36.77)	0.330
Combined DLP (*n*, %)	192(30.87)	425(26.27)	0.029
Combined CVD (*n*, %)	233(37.46)	448(27.69)	<0.001
Combined Cancer (*n*, %)	104(16.72)	267(16.50)	0.901

Abbreviation: ALT, Alanine Aminotransferase; AST, Aspartate Aminotransferase; BMI, Body Mass Index; CAP, controlled attenuation parameter; CVD, Cardiovascular Diseases; DBP, Diastolic Blood Pressure; DLP, Dyslipidemia; GGT, Gamma Glutamyl Transferase; HbA1c, Glycated hemoglobin A1; HS-CRP, high-sensitivity C-reactive protein; HTN, Hypertension; LSM, Liver Stiffness Measurements; LDH, Lactate Dehydrogenase; M, Median; PA, Physical Activity; SD, standard deviations; SBP, Systolic Blood Pressure; T2D, Type 2 diabetes; TC, Total Cholesterol; TG, Total Triglycerides; WC, Waist Circumference;

Table S2. Weighted prevalence of liver fibrosis among participants[*n*(weighted prevalence, (95% CI)]

Group	T2D(*n*=622)	No-T2D(*n*=1618)
Any degree of liver fibrosis	131(23.04%, 95% CI: 17.86% - 29.20%)	150(6.70%, 95% CI:5.10% - 8.16%)
LSM (kPa)		
8.2~	39(7.21%, 95% CI: 4.49% -11.38%)	57(2.05%, 95% CI: 1.35% - 3.10%)
9.7~	51(8.44%, 95% CI: 5.58% - 12.57%)	55(2.55%, 95% CI: 1.69% - 3.83%)
13.6≥	41(7.39%, 95%CI: 4.24% - 12.59%)	38(2.10%, 95% CI: 1.15% - 3.81%)

Abbreviation: 95%CI, 95% confidence intervals; Liver Stiffness Measurements; T2D, type 2 diabetes

Table S3. Association of weight changes across three lifespans with liver fibrosis risk among T2D patients

Liver Fibrosis	Weight change patterns			
	Stable Non-obese	Weight Loss	Weight Gain	Stable Obese
	Reference	OR (95%CI)	OR (95%CI)	OR (95%CI)
BMI $_{age\ 25}$ to BMI $_{10\ years\ prior}$				
Events /Total	42/278	0/11	73/276	16/57
Model 1	1.00	-	2.02(1.32,3.09)	2.19(1.13,4.26)
Model 2	1.00	-	2.05(1.34,3.14)	2.04(1.04,4.01)
Model 3	1.00	-	1.86(1.17,2.95)	1.89(1.01,3.91)
BMI $_{age\ 25}$ to BMI $_{2017\ -\ 2018\ cycle}$	Stable Non-obese	Weight Loss	Weight Gain	Stable Obese
	Reference	OR (95%CI)	OR (95%CI)	OR (95%CI)
Events /Total	32/263	0/16	83/291	16/52
Model 1	1.00	-	2.88(1.84,4.51)	3.21(1.60,6.43)
Model 2	1.00	-	2.95(1.87,4.65)	3.18(1.56,6.49)
Model 3	1.00	-	2.53(1.55,4.11)	2.89(1.35,6.21)
BMI $_{10\ years\ prior}$ to BMI $_{2017\ -\ 2018\ cycle}$	Stable Non-obese	Weight Loss	Weight Gain	Stable Obese
	Reference	OR (95%CI)	OR (95%CI)	OR (95%CI)

Events /Total	24/213	8/66	18/76	81/267
Model 1	1.00	1.09(0.46,2.55)	2.44(1.24,4.82)	3.43(2.08,5.65)
Model 2	1.00	1.06(0.42,2.40)	2.47(1.23,4.90)	3.49(2.14,5.89)
Model 3	1.00	1.00(0.39,2.31)	2.06(0.98,4.22)	3.13(1.84,5.48)

Abbreviation: BMI, body mass index; OR, odds ratios; 95%CI, 95% confidence intervals; T2D, type 2 diabetes

Model 1: crude model.

Model 2: Adjusted for sex and age.

Model 3: Adjusted for sex, age, education, marital status, smoking, alcohol consumption, physical activity, SBP, DBP, WC, ALT, AST, GGT, LDH, hs-CRP, Uric acid to creatinine ratio, Albumin to globulin ratio TC, TG, Platelet count, dyslipidemia, hypertension, CVD, and cancer.

Table S4. Association of weight changes across three lifespans with liver fibrosis risk among no-T2D participants

Liver Fibrosis	Weight change patterns			
	Stable Non-obese	Weight Loss	Weight Gain	Stable Obese
	Reference	OR (95%CI)	OR (95%CI)	OR (95%CI)
BMI $_{age\ 25}$ to BMI $_{10\ years\ prior}$				
Events /Total	83/1167	1/12	57/375	9/64
Model 1	1.00	1.19(0.15, 9.31)	2.34(1.63, 3.35)	2.14(1.02, 4.78)
Model 2	1.00	1.16(0.42, 9.18)	2.27(1.58, 3.26)	2.09(1.00, 4.41)
Model 3	1.00	0.08(0.01, 11.48)	2.24(1.51, 3.33)	1.89(0.83, 4.30)
BMI $_{age\ 25}$ to BMI $_{2017\ -\ 2018\ cycle}$	Stable Non-obese	Weight Loss	Weight Gain	Stable Obese
	Reference	OR (95%CI)	OR (95%CI)	OR (95%CI)
Events /Total	64/1033	1/24	76/509	9/52
Model 1	1.00	0.66(0.08, 4.95)	2.66(1.87, 3.78)	3.17(1.48, 6.79)
Model 2	1.00	0.58(0.08, 4.14)	2.83(1.99, 4.04)	3.46(1.59, 7.51)
Model 3	1.00	0.02(0.001, 1.52)	3.32(2.20, 5.01)	3.28(1.40, 7.67)
BMI $_{10\ years\ prior}$ to BMI $_{2017\ -\ 2018\ cycle}$	Stable Non-obese	Weight Loss	Weight Gain	Stable Obese
	Reference	OR (95%CI)	OR (95%CI)	OR (95%CI)

Events /Total	58/949	7/108	26/230	59/331
Model 1	1.00	1.07(0.47, 2.40)	1.96(1.20, 3.19)	3.33(2.26, 4.91)
Model 2	1.00	0.98(0.44, 2.22)	2.20(1.34, 3.62)	3.28(2.08, 4.69)
Model 3	1.00	0.88(0.36, 2.13)	1.79(0.45, 4.41)	2.63(1.40, 4.42)

Abbreviation: BMI, body mass index; OR, odds ratios; 95%CI, 95% confidence intervals;

Model 1: crude model.

Model 2: Adjusted for sex and age.

Model 3: Adjusted for sex, age, education, marital status, smoking, alcohol consumption, physical activity, SBP, DBP, WC, ALT, AST, GGT, LDH, hs-CRP, Uric acid to creatinine ratio, Albumin to globulin ratio TC, TG, Platelet count, dyslipidemia, hypertension, CVD, and cancer.

Table S5. Association of absolute weight change patterns with liver fibrosis risk among T2D patients

Liver Fibrosis	Weight change within 2.5 kg	Weight loss ≥2.5 kg OR (95%CI)	Weight gain ≥2 .5 kg and <10.0 kg OR (95%CI)	Weight gain ≥10 kg and <20 kg OR (95%CI)	Weight gain ≥20 kg OR (95%CI)
BMI age 25 to BMI 10 years prior					
Events/Total	10/56	2/26	20/124	34/187	65/229
Model 1	1.00	0.38 (0.06,1.60)	0.88(0.39,2.10)	1.02(0.48,2.32)	1.82(0.90,4.02)
Model 2	1.00	0.40(0.06,1.66)	0.98(0.43,2.34)	1.13(0.53,2.58)	1.20(0.98,4.44)
Model 3	1.00	0.38(0.06,1.63)	1.04(0.45,2.56)	1.12(0.51,2.61)	1.73(0.82,3.91)
BMI age 25 to BMI 2017 - 2018 cycle					
Events/Total	11/39	4/65	12/98	28/161	76/259
Model 1	1.00	0.17(0.04,0.53)	0.36(0.14,0.90)	0.54(0.24,1.23)	1.06(0.51,2.32)
Model 2	1.00	0.16(0.04,0.52)	0.37(0.14,0.94)	0.55(0.25,1.30)	1.13(0.54,2.51)
Model 3	1.00	0.19(0.05,0.67)	0.47(0.18,1.31)	0.62(0.26,1.59)	1.32(0.59,3.22)
BMI 10 years prior to BMI 2017 - 2018 cycle					
Events/Total	15/92	47/265	28/139	22/79	19/47
Model 1	1.00	1.11(0.60,2.15)	1.29(0.66,2.64)	1.98(0.95,4.22)	3.48(1.57,7.89)
Model 2	1.00	1.08(0.58,2.10)	1.30(0.66,2.65)	1.95(0.93,4.18)	3.47(1.54,7.97)

Model 3	1.00	0.98(0.50,1.98)	1.29(0.63,2.73)	2.13(0.98,4.77)	2.94(1.24,7.07)

Abbreviation: BMI, body mass index; OR, odds ratios; 95%CI, 95% confidence intervals; T2D, type 2 diabetes

Model 1: crude model.

Model 2: Adjusted for sex and age.

Model 3: Adjusted for sex, age, education, marital status, smoking, alcohol consumption, physical activity, SBP, DBP, WC, ALT, AST, GGT, LDH, hs-CRP, Uric acid to creatinine ratio, Albumin to globulin ratio TC, TG, Platelet count, dyslipidemia, hypertension, CVD, and cancer.

Table S6. Subgroup analysis of weight change across three lifespans and liver fibrosis risk among T2D patients

Liver Fibrosis	Stable Normal<br>Reference	Weight Loss<br>OR (95%CI)	Weight Gain<br>OR (95%CI)	Stable Obese<br>OR (95%CI)	*p*-interaction
BMI $_{age\ 25}$ to BMI $_{10\ years\ prior}$					
Sex					0.766
Males	1.00	-	1.94(1.06,3.55)	1.52(0.51,4.51)	
Female	1.00	-	1.88(0.87,4.06)	2.42(0.85,6.89)	
2017 - 2018 cycle Age (years)					0.942
⩾65	1.00	-	1.63(0.87,3.08)	2.08(0.64,6.73)	
<65	1.00	-	2.19(1.10,4.36)	2.00(0.78,5.15)	
Disease duration(years)					0.703
⩾10	1.00	-	2.03(1.03,4.00)	1.82(0.59,5.65)	
<10	1.00	-	1.67(0.74,3.78)	1.79(0.54,5.94)	
BMI $_{age\ 25}$ to BMI $_{2017\ -\ 2018\ cycle}$					
Sex					0.617
Males	1.00	-	3.26(1.73,6.14)	2.95(0.95,9.17)	
Female	1.00	-	1.85(0.82,4.18)	2.61(0.86,7.93)	

2017 - 2018 cycle Age (years)					0.999
⩾65	1.00	-	2.63(1.36,5.09)	2.87(0.87,9.45)	
<65	1.00	-	2.80(1.32,5.92)	3.23(1.15,9.07)	
Disease duration(years)					0.037
⩾10	1.00	-	4.00(1.75,9.10)	4.88(1.38,17.35)	
<10	1.00	-	1.85(0.86,4.00)	1.81(0.57,5.76)	
BMI 10 years prior to BMI 2017 - 2018 cycle					
Sex					0.600
Males	1.00	0.77(0.25,2.35)	2.38(0.89,6.40)	3.64(1.79,7.42)	
Female	1.00	1.80(0.38,8.40)	1.68(0.53,5.31)	2.73(1.07,6.95)	
2017 - 2018 cycle Age (years)					0.414
⩾65	1.00	1.47(0.50,4.32)	3.41(1.22,9.53)	2.98(1.39,6.42)	
<65	1.00	0.50(0.10,2.51)	1.52(0.52,4.43)	3.56(1.59,7.95)	
Disease duration(years)					0.489
⩾10	1.00	1.19(0.31,4.50)	2.66(0.94,7.58)	3.55(1.54,8.21)	
<10	1.00	0.62(0.16,2.43)	0.86(0.15,2.53)	2.64(1.09,6.39)	

Abbreviation: BMI, body mass index; OR, odds ratios; 95%CI, 95% confidence intervals; T2D, type 2 diabetes

This model has adjusted for sex, age, education, marital status, smoking, alcohol consumption, physical activity, SBP, DBP, WC, ALT, AST, GGT, LDH, hs-CRP, Uric acid to creatinine ratio, Albumin to globulin ratio TC, TG, Platelet count, dyslipidemia, hypertension, CVD, and cancer.

Table S7. Population attributable fractions (PAF) for population counterfactuals of fibrosis among T2D patients

Scenario	Definition	PAF (%), 95% CI of Total Population
Weight loss	If those who gained weight from early adulthood instead loss weight during middle and late adulthood	60.71(39.75, 64.83)
Weight maintenance	If those who gained weight during adulthood had not gained weight	58.55(37.05, 72.71)
Partial prevention	If the total population maintained non-obese (BMI < 30 kg/m^2) during the life course	53.70(30.24, 69.28)
Comprehensive prevention	If the total population had a normal BMI (BMI < 25 kg/m^2) cross adulthood	64.96(-5.13, 88.32)

Abbreviation: BMI, body mass index; PAF, population attributable fractions;

Table S8. Sensitivity analysis of the association between weight change and liver fibrosis in T2D patients

Outcome	Stable Non-obese (Reference)	Weight Loss OR (95%CI)	Weight Gain OR (95%CI)	Stable Obese OR (95%CI)
Mild liver fibrosis				
BMI $_{age\ 25}$ to BMI $_{10\ years\ prior}$				
Events/Total	13/249	0/11	22/225	4/45
Model 1	1.00	-	1.97(0.97,4.01)	1.77(0.55,5.70)
Model 2	1.00	-	2.02(0.99,4.13)	1.44(0.44,4.73)
Model 3	1.00	-	2.20(0.99,4.88)	1.64(0.46,5.90)
BMI $_{age\ 25}$ to BMI $_{2017\ -\ 2018\ cycle}$				
Events/Total	13/244	0/16	22/230	4/40
Model 1	1.00	-	1.88(0.92,3.83)	1.97(0.61,6.39)
Model 2	1.00	-	1.82(0.88,3.75)	1.67(0.50,5.59)
Model 3	1.00	-	1.75(0.79,3.91)	1.83(0.51,6.64)
BMI $_{10\ years\ prior}$ to BMI $_{2017\ -\ 2018\ cycle}$				
Events/Total	9/198	4/62	4/62	22/208
Model 1	1.00	1.45(0.43,4.88)	1.45(0.44,4.86)	2.48(1.11,5.54)

Model 2	1.00	1.43(0.42,4.84)	1.35(0.40,4.61)	2.41(1.07,5.43)
Model 3	1.00	1.48(0.41,5.29)	1.17(0.29,4.70)	2.66(1.07,6.60)
Advanced liver fibrosis				
BMI $_{\text{age 25}}$ to BMI $_{\text{10 years prior}}$				
Events/Total	29/278	0/11	51/276	12/57
Model 1	1.00	-	1.95(1.19,3.18)	2.29(1.09,4.82)
Model 2	1.00	-	1.95(1.20,3.19)	2.28(1.07,4.85)
Model 3	1.00	-	1.65(0.98,2.81)	1.96(0.87,4.41)
BMI $_{\text{age 25}}$ to BMI $_{\text{2017 - 2018 cycle}}$				
Events/Total	19/263	0/16	61/291	12/52
Model 1	1.00	-	3.41(1.91,5.88)	3.85(1.74,8.54)
Model 2	1.00	-	3.57(2.06,6.21)	4.15(1.83,9.39)
Model 3	1.00	-	3.06(1.71,5.49)	3.64(1.52,8.74)
BMI $_{\text{10 years prior}}$ to BMI $_{\text{2017 - 2018 cycle}}$				
Events/Total	15/213	4/66	14/76	59/267
Model 1	1.00	0.85(0.27,2.66)	2.98(1.36,6.52)	3.74(2.06,6.82)
Model 2	1.00	0.82(0.26,2.57)	3.14(1.42,6.91)	3.89(2.13,7.12)

Model 3	1.00	0.72(0.23,2.30)	2.66(1.17,6.05)	3.30(1.73,6.29)
Cirrhosis				
BMI $_{age\ 25}$ to BMI $_{10\ years\ prior}$				
Events/Total	14/278	0/11	22/276	5/57
Model 1	1.00	-	1.63(0.82,3.26)	1.81(0.63,5.25)
Model 2	1.00	-	1.65(0.83,3.30)	1.74(0.59,5.12)
Model 3	1.00	-	1.50(0.72,3.14)	1.41(0.42,4.76)
BMI $_{age\ 25}$ to BMI $_{2017\ -\ 2018\ cycle}$				
Events/Total	10/263	0/16	26/291	5/52
Model 1	1.00	-	2.48(1.17,5.25)	2.69(0.88,8.23)
Model 2	1.00	-	2.57(1.21,5.49)	2.75(0.88,8.65)
Model 3	1.00	-	2.34(1.06,5.19)	2.34(0.65,8.46)
BMI $_{10\ years\ prior}$ to BMI $_{2017\ -\ 2018\ cycle}$				
Events/Total	8/213	2/66	6/76	25/267
Model 1	1.00	0.80(0.17,3.87)	2.20(0.74,6.55)	2.65(1.17,6.00)
Model 2	1.00	0.77(0.16,3.73)	2.27(0.75,6.83)	2.73(1.20,6.21)
Model 3	1.00	0.72(0.15,3.59)	2.04(0.65,6.35)	2.45(1.06,5.92)

Abbreviation: BMI, body mass index; OR, odds ratios; 95%CI, 95% confidence intervals

Model 1: Crude model.

Model 2: Adjusted for sex and age.

Model 3: Adjusted for sex, age, education, marital status, smoking, alcohol consumption, physical activity, SBP, DBP, WC, ALT, AST, GGT, LDH, hs-CRP, Uric acid to creatinine ratio, Albumin to globulin ratio TC, TG, Platelet count, dyslipidemia, hypertension, CVD, and cancer.

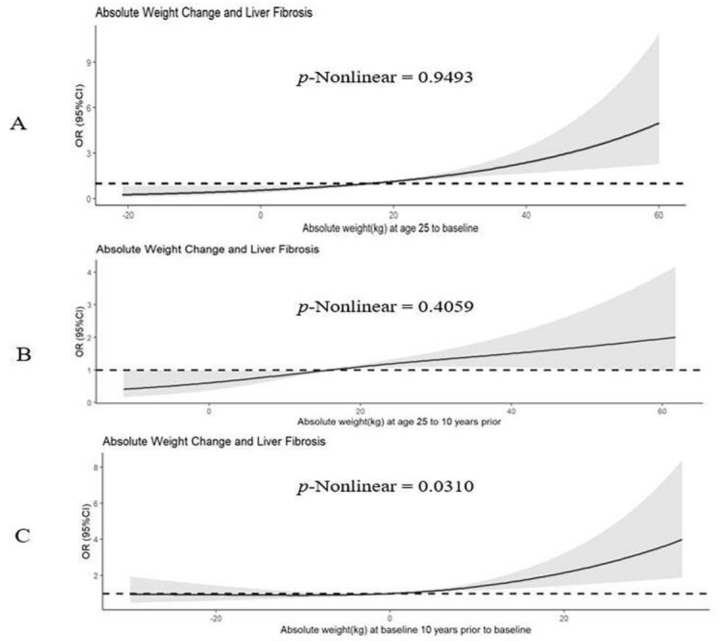

Figure S1. Dose-response curves of absolute weight change associated with liver fibrosis risk among T2D patients.
